# Interspaced Repeat Sequences Confer the Regulatory Functions of AtXTH10, Important for Root Growth in Arabidopsis

**DOI:** 10.3390/plants8050130

**Published:** 2019-05-16

**Authors:** Jun Cao, Yueqing Lv, Xiang Li

**Affiliations:** Institute of Life Sciences, Jiangsu University, Zhenjiang 212013, China; 19939707750@163.com (Y.L.); xiang.li2@simceredx.com (X.L.)

**Keywords:** IRSs, promoter, gene expression regulation, xyloglucan endotransglucosylase/hydrolase, root development

## Abstract

An interspaced repeat sequence (IRS) is a unique sequence similar to prokaryotic CRISPR in structure. In this study, 1343 IRSs were identified in the Arabidopsis genome. Functional annotation of the IRS-related genes showed that they were associated with various growth and development processes. More than 30% of the IRSs were located in promoter regions. Deletion of some IRSs affected promoter activity, suggesting their roles in the regulation of gene expression. Next, the function of the *AtXTH10* gene was further analyzed, and the expression of this gene was regulated by IRSs in its promoter region. Transgenic and mutant plants analysis indicated that the *AtXTH10* gene was associated with root development by affecting cell wall structure. Moreover, the expression profiles of some key genes involved in root development signaling pathways were also affected by *AtXTH10*. These results suggest that IRSs could be involved in regulating the expression of genes with important roles in plant development.

## 1. Introduction

A repeat sequence is a special arrangement form of DNA. Prokaryotic and eukaryotic genomes contain many different types of repetitive DNA sequences, such as tandem repeats and inverted repeats [1], that play important roles in genome evolution, chromosome rearrangement, and regulation of expression [2,3]. Transcription is an important regulatory step in gene expression. The promoter possesses a variety of *cis*-elements, which play very important roles in transcription [4]. Therefore, it is important to study the functional sequence of a promoter to investigate the gene expression mechanism. The promoter is also an important component of the engineering vector. Therefore, understanding the molecular nature of the promoter is an important way to provide highly efficient expression vectors [5].

Interspaced repeat sequences (IRSs) are sequences with unique structures in the genome, in which the repeat units are separated by interval sequences. The sequences of the repeat units are highly conserved and are separated by different intervals or spacer sequences within an IRS. Different repeat units usually occur between different IRSs. IRSs are structurally similar to the CRISPR (clustered regularly interspaced short palindromic repeat) sequences in bacteria, which are important for RNA-guided adaptive immunity system with the CRISPR-associated (Cas) protein [6]. This system has been widely used for genomic engineering and editing in many studies [7,8]. *Cas* genes are present in almost all archaea and many bacteria, and this system only exists in these organisms [9,10]. In other words, *Cas* genes might have disappeared from eukaryotes during evolution. No studies have been conducted on the CRISPR similar IRS sequences in eukaryotes.

As plant-specific Ca^2+^ sensors, calcineurin B-like proteins (CBLs) can interact with CBL-interacting protein kinases (CIPKs) and play key roles in plant growth and development and various abiotic stress responses [11,12]. CBLs have been identified in Arabidopsis and other species. CBL9 is involved in abscisic acid biosynthesis during seed germination [13]. CBLs also affect pollen germination and pollen tube growth by controlling K^+^ homeostasis [14,15]. CBL1 positively regulates the salt and drought responses of plants [16]. Moreover, overexpression of the *CBL5* gene endows the transgenic plants with osmotic or drought resistance [17].

Xyloglucan endotransglucosylase/hydrolase (XTH) widely occurs in various tissues and cells of plants. It belongs to a member of the glycoside hydrolase GH16 family. According to its sequence and function, the XTH protein can be divided into three major classes (I, II, and III). Among them, classes I and II have both xyloglcan endotransglycosylase (XET) and xyloglucan endohydrolase (XEH) activities, while class III proteins only have XEH activity. XTH modifies the cellulose–xyloglucan complex structure of the plant cell wall by catalyzing the breakage and relinking of the xyloglucan molecule [18]. It first combines with a substrate by hydrolysis to form a covalent intermediate of the glycosyl enzyme. Then, the enzyme is transferred to the reductive end of the polysaccharide residue and performs the glycosyl reaction (XET activity), or the enzyme hydrolyzes glycosylated water molecules (XEH activity) [19]. Studies have shown that *XTH* genes are involved in multiple growth and development processes in plants. The *CaXTH1* gene of chickpea can be efficiently induced by hormones in growth tissues and plays an important role in elongation of vascular cells [20]. The rose ethylene-responsive *RbXTH1* and *RbXTH2* genes have different expression levels during different flower developmental stages, indicating that they may play diverse roles in the growth and development of rose flowers and petal abscission [21]. The expression levels of some *XTH* genes change under mechanical stimulation or aluminum and hypoxic stressors [22,23,24]. Brassinosteroids (BR) regulate activity of the XTH enzyme and increase ductility of the cell wall. In Arabidopsis, the expression levels of the *AtXTH22* and *AtXTH24* increase significantly after treatment with BR, which could be further related with cell wall elongation. In contrast, their expression levels decrease significantly in *dwf1* mutant plants [25]. Treating Arabidopsis seedlings with gibberellin increases transcription of the *AtXTH21* gene, indicating that *AtXTH21* is affected by this hormone [26].

For the first time, IRSs were identified in Arabidopsis, and their putative role in controlling gene expression was demonstrated here. Manipulation of an IRS located at the AtXTH10 promoter region using mutants and transgenic plants suggested that IRSs can affect transcriptional activity of the promoter, and that *AtXTH10* regulates root growth and development by altering plant cell wall structure. These results indicate that eukaryotic IRSs may be involved in the growth and development of plants by regulating the expression of key genes.

## 2. Materials and Methods

### 2.1. Identification of Arabidopsis IRS and Functional Annotation of the IRS-Related Genes

All previously sequenced Arabidopsis BAC sequences were obtained from TAIR (The Arabidopsis Information Resource: www.arabidopsis.org) and submitted to the CRISPRFinder tool [27] to identify the IRS structures with default parameters. Next, all predicted IRSs (Table S1) were located to the genome according to their sequences and BAC clone information. In this study, an IRS-related gene was defined as a gene containing IRSs in its sequence (including the promoter region). Functional annotation of the IRS-related genes was performed with a Gene Ontology (GO) enrichment analysis server (http://geneontology.org) [28].

### 2.2. Functional Identification of the IRSs in the CR6 and CR12 Promoters

In our previous study, 13 gene promoters were analyzed and their transcriptional activity was observed by mutating some IRSs. The results showed that about half of the promoter activity was affected by its IRS sequence. Two of the most representative promoters, CR6 and CR12, were selected for further analysis. The IRSs on these two promoters play completely opposite roles in their activity. To isolate the CR6 promoter (that is, the promoter of the *AT2G14620* (*AtXTH10*) gene) and to develop the expression vectors lacking the different IRS, specific primers (CR6-1, CR6-2, CR6-4, and CR6-6) were designed (see details at Table S2) using the Primer3 program [29,30] following default parameters. The CR6-1/CR6-2 primers were used to isolate the wild type promoter CR6. Then, using the CR6 promoter as a template, the CR6-1/CR6-4, CR6-1/CR6-6, and CR6-1/CR6-8 primers were used to develop the other vectors (CR6m1, CR6m2, and CR6m3), which lack different IRSs or spacers, respectively. In the same way, the CR12 promoter (that is, the promoter of *AT4G01420* (*CBL5*) gene) isolation and deletion were performed with specific primers (CR12-1, CR12-2, CR12-a, CR12-b, CR12-c, and CR12-3) (Table S2). After all promoters or their mutants were confirmed to be correct by sequencing, they were inserted into the pBI121 vector, in which the 35S promoter was cleaved by restriction endonucleases (*Hin*dIII and *Bam*HI) to construct different expression vectors. Agrobacteria infiltration [31] in tobacco leaves and GUS measurements [32] were used to determine the activities of these promoters.

### 2.3. Vector Construction and Subcellular Localization of the AtXTH10 Protein

cDNA was synthetized by reverse transcription using 0.5 μg RNA extracted with the TRIzol^®^ total RNA extraction kit (Sangon, Shanghai, China), 2 μL of 4×gDNA Wiper Mi and Nuclease-free H_2_O were added until the total reaction volume (8 µL) was met. Mixtures were incubated in a GeneAmp^®^ PCR System 9700 (Applied Biosystems, Foster City, CA, USA) for 2 min at 42 °C. Further, 2 μL of 5× HiScript II Q RT SuperMix IIa was added and reverse transcription reaction was run in a GeneAmp^®^ PCR System 9700 (Applied Biosystems, USA) for 10 min at 25 °C followed by 30 min at 50 °C, and 5 min at 85 °C. The primers (AtXTH10-F; AtXTH10-R) (Table S2) were designed as described above and used for isolating open reading frame of the *AtXTH10* gene using the synthetized cDNA as the template. The gene was sequenced for verification and then inserted into the pBI121-eGFP vector (named pBI121-AtXTH10-eGFP), which was further transformed into *Agrobacterium tumefaciens* EHA105 for transient expression [33]. The pBI121-eGFP vector was modified based on the pBI121 vector, that is, the GUS gene of the pBI121 vector was replaced with the eGFP gene. The inner epidermis of onion was placed on 1/2MS (1.5% sucrose) solid medium for transient expression [34]. *A. tumefaciens* EHA105 containing the pBI121-AtXTH10-eGFP vector constructed above was cultured as described previously [31]. The cells were then resuspended in a solution containing 10 mM Mes (pH 5.5), 10 mM MgSO_4_, and 100 mM acetosyringone, and the OD_600_ of the suspension was adjusted to 0.5–0.8 before infiltration. The liquid Agrobacterium bacterial solution was dripped onto the inner epidermis of the onion for about 30 min using a pipette. The excess was washed off with filter paper. Inoculated tissues were incubated at 28 °C under dark conditions for 2 days. After cocultivation period, onion epidermis tissues were rinsed gently twice with 1×PBS (phosphate buffered saline) solution (pH7.0) and one time with sterile water, and further placed on glass slides for fluorescence microscopy.

### 2.4. Obtaining Transgenic AtXTH10 and Mutant Plants

*AtXTH10* gene cloning and vector construction were described above. *A. tumefaciens* EHA105 containing the 35S::AtXTH10 vector was used to transform *A. thaliana* (Col) plants using the floral dip method [35]. Kanamycin (35 μg/mL)-resistant transformants (T0) were screened by polymerase chain reaction (PCR) using genomic DNA as the template; the forward primer (35S-1) is located at the 35S promoter and the reverse primer (AtXTH10-R) is located at the AtXTH10 gene. Homozygotes (T2) obtained from two generations of self-bred transgenic plants (T0) were used for the phenotypic analysis. T-DNA insertion mutant (SALK_1235) of the *AtXTH10* gene was obtained from the ABRC (Arabidopsis Biological Resource Center, http://abrc.osu.edu). In this mutant, one T-DNA was inserted into the first exon of the *AtXTH10* gene (Figure S1).

### 2.5. Scanning Electron Microscopic Observations

To visualize the changes in root microstructure in the wild type (Col), mutant (AtXTH10m) and overexpressing (AtXTH10-OE) plants, the samples were first fixed in solutions containing 0.05 M potassium phosphate (pH 7.0), 4% (w/v) paraformaldehyde, and 0.02% Triton X-100 in vacuum for 3–5 h to ensure that the solutions penetrated into the tissues. The fixed samples were left in the same solutions at 4 °C overnight, and then rinsed several times with 0.05 M potassium phosphate (pH 7.0) and distilled water. The samples were dehydrated through a graded ethanol series at room temperature. The tissue samples were dried in liquid carbon dioxide. Each root tissue was mounted on conductive tape, and sputtered with 10 nm JFC-1600 ion-plated gold film for 120 s. The structures were observed with a JSM-6360LV scanning electron microscope and photographed [36,37].

### 2.6. Detection the Relative Expression Levels of Known Root Development-Related Genes by Reverse Transcription Quantitative Real-Time Polymerase Chain Reaction (RT-qPCR) in the Col, AtXTH10m and AtXTH10-OE Plants

To detect signal transduction of the *AtXTH10* gene in response to root development, the expression patterns of 24 known genes (Table S3) that affect root growth of Arabidopsis were further examined in the wild type (Col), mutant (AtXTH10m), and overexpressing (AtXTH10-OE) plants. Plant seedlings were grown in a greenhouse at 24 °C under a 14 h photoperiod. Total RNA of 1-week-old seedlings was extracted with the TRIzol^®^ total RNA extraction kit (Sangon, Shanghai, China). RNA yield was determined using a NanoDrop 2000 spectrophotometer (Thermo Scientific, Waltham, MA, USA), and integrity was evaluated using agarose gel electrophoresis followed by staining with ethidium bromide (EB). A 0.5 μg aliquot of RNA, 2 μL of 4× gDNA Wiper Mix, and Nuclease-free H_2_O was added to 8 μL of total reaction volume and incubated in a GeneAmp^®^ PCR System 9700 (Applied Biosystems, Foster City, CA, USA) for 2 min at 42 °C. Further, 2 μL of 5× HiScript II Q RT SuperMix IIa was added and reverse transcription have run in a GeneAmp^®^ PCR System 9700 (Applied Biosystems, USA) for 10 min at 25 °C followed by 30 min at 50 °C, and for 5 min at 85 °C. The 10 μL RT reaction mix was then diluted ×10 in nuclease-free water and held at −20 °C. Real-time PCR was performed using the LightCycler^®^ 480 II Real-time PCR Instrument (Roche, Basel, Switzerland) with a 10 μL PCR reaction mixture that included 1 μL of cDNA, 5 μL of 2× QuantiFast^®^ SYBR^®^ Green PCR Master Mix (Qiagen, Hilden, Germany), 0.2 μL of each forward and reverse primer (10 μM), and 3.6 μL of nuclease-free water. Reactions carried out in a 384-well optical plate (Roche) for 40 cycles, each cycle consisting in 10 s at 95 °C followed by 30 s at 60 °C. An initial step at 95 °C for 5 min was also considered. Nontemplate controls (NTC) were used to monitor for potential contamination within the RT-qPCR reagents. Three biological replicates were used for the analysis. A melting curve analysis was performed to validate specific generation of the expected PCR product. The primer sequences (Table S3) were designed as described above. The mRNA expression levels were normalized to those of the Arabidopsis *actin2* gene (*AT3G18780.1*) [38] and were calculated using the 2^−ΔΔCt^ method [39].

## 3. Results and Discussion

### 3.1. Identification of IRSs and Functional Annotation of the IRS-Related Genes

**Figure 1:**
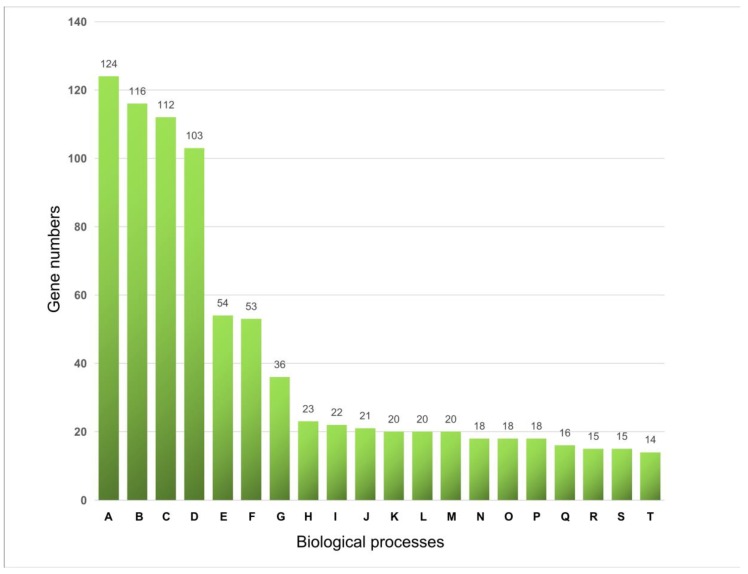
Gene Ontology (GO) functional enrichment analysis of the interspaced repeat sequence (IRS)-related genes in Arabidopsis. GO enrichment analysis was performed using the online server (http://geneontology.org). Approximately 914 mapped IDs and results only for uncorrected *p* < 0.05 were displayed. The top 20 biological processes are shown here. A: developmental process; B: anatomical structure development; C: multicellular organismal process; D: multicellular organism development; E: macromolecule biosynthetic process; F: cellular macromolecule biosynthetic process; G: cellular amide metabolic process; H: growth; I: cellular carbohydrate metabolic process; J: cell growth; K: plant organ morphogenesis; L: chemical homeostasis; M: secondary metabolic process; N: polysaccharide metabolic process; O: cellular polysaccharide metabolic process; P: pollen development; Q: plant epidermis development; R: root morphogenesis; S: positive regulation of metabolic process; T: cellular carbohydrate biosynthetic process.

The entire genome sequence of *A. thaliana* was first analyzed and about 1343 IRS structures were identified (Table S1). A GO enrichment analysis was performed to investigate the functional relevance of IRS-related genes present in this genome. The results revealed that most IRS-related genes were involved in molecular functions, such as binding, monooxygenase activity, oxygen binding, and translation factor activity. In addition, biological processes, such as developmental processes, structural development, organism development, and macromolecule biosynthetic processes, were common in the IRS-related genes (Figure 1). These findings suggest that IRSs may play roles in various developmental processes in Arabidopsis.

### 3.2. IRSs Confer a Regulatory Function of Gene Expression

**Figure 2:**
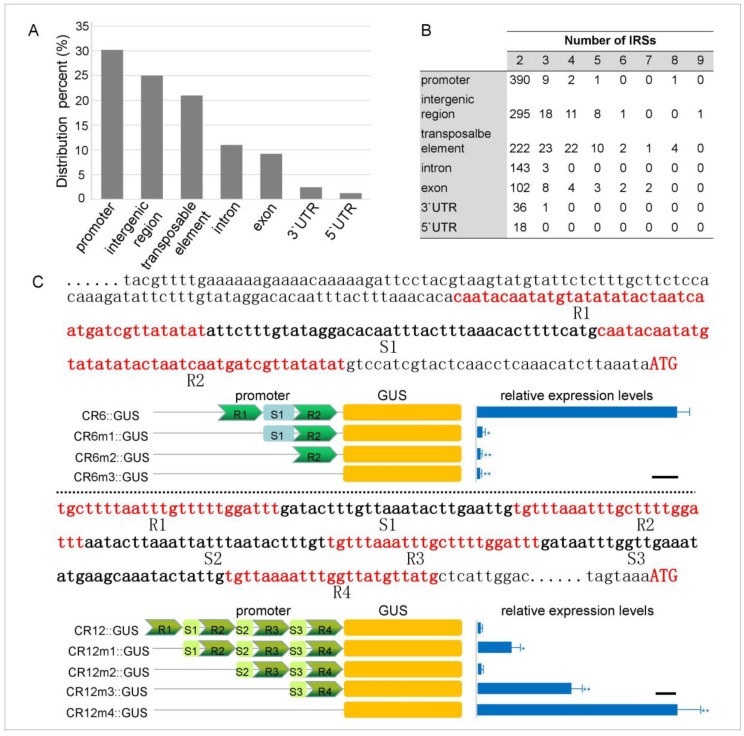
Distribution characteristics (**A**) and number (**B**) of Arabidopsis IRSs and regulation function analysis of IRSs in CR12 and CR6 promoters (**C**). Different repeat units and spacer sequences are identified in the form of R and S, respectively. The numbers behind R and S are helpful to distinguish them. The scale bar of GUS activity is 100 (4-MU pmol/mg protein/min). Significance was tested relative to each wild type using *t*-test. Significance of * *p* < 0.05 and ** *p* < 0.01. Error bar: standard deviation.

Next, the genomic locations of these IRSs were determined. The results indicated that more than 30% of the sequences were located in the promoter regions, accounting for the maximum number of all location types (Figure 2A). The second location-dense space was the intergenic region, representing ~25% of all IRSs. About one-fifth of the IRSs were located in or near the transposable element. A very small number of IRS sequences were located in the 5’-untranslated region (UTR) and the 3’-UTR (Figure 2A). Moreover, the number of repeat units was nonuniformly distributed in all identified IRSs, that is, 89.79% of the IRSs contained two repeat units and a maximum of nine repeat units appeared in an intergenic region (Figure 2B). In bacteria, 63% of the IRS similar structures (CRISPRs) have fewer than five repeat units. Some CRISPR loci are repeated up to 250 times [40]. This suggests that the number of repeat IRS units in Arabidopsis is much lower than that in bacteria. The promoter is a key gene expression regulatory region [41]. As more than 30% of the IRSs were located in the gene promoter region, it is considered whether these IRSs conferred a gene expression regulatory function. To answer this question, 13 promoters containing the IRSs were randomly selected to evaluate the effect of deletions in our previous study. Through comparing the expressive activity of the wild type promoter and the deleted IRSs promoter, it is determined whether the IRSs can regulate the promoter activity. The results indicated that some IRSs affected the promoter activity, whereas others had little effect. Two promoters were examined in detail (Figure 2C). One was the promoter (named CR6) of the *AT2G14620* gene (encoding the xyloglucan endotransglucosylase/hydrolase 10 (AtXTH10) protein), and the other was the promoter (named CR12) of the *AT4G01420* gene [encoding the calcineurin B-like 5 (CBL5) protein]. The CR6 promoter contained two repeats (R1 and R2), and deleting the repeats resulted in a sharp decrease in the promoter activity, indicating that these IRSs could act as *cis*-elements and enhance promoter activity (Figure 2C). There were four repeat units in the CR12 promoter. When R1, R3, and R4 were sequentially deleted, promoter activity was gradually enhanced, indicating that the presence of these three repeat units partially inhibited activity of the CR12 promoter. However, when R2 was deleted, promoter activity decreased, suggesting that the effect of the R2 repeat is just the opposite of the other three repeats (R1, R3, and R4) (Figure 2C). These results suggest that the IRSs on the Arabidopsis promoter region act as *cis*-elements and play an important role regulating gene expression.

### 3.3. The AtXTH10 Gene Regulates the Growth and Development of Roots

**Figure 3:**
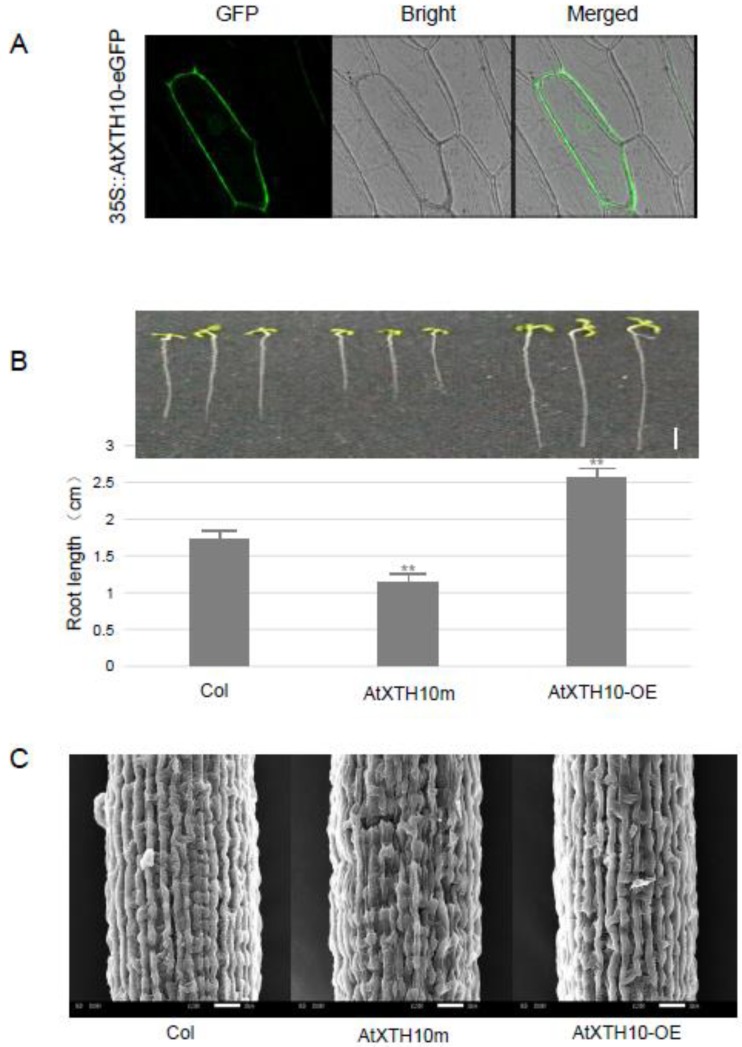
Subcellular localization of AtXTH10 protein (**A**), phenotypic analysis of *AtXTH10* gene affecting root development (scale bar stands for 0.5 cm) (**B**), and scanning electron microscope observation of root microstructure (scale bar is 10 μm) (**C**). Significance was tested relative to the wild type (Col) using *t*-test. Significance of ** *p* < 0.01. Error bar: standard deviation.

As described above, IRSs regulated the expression of the *CBL5* and *AtXTH10* genes. Overexpression of the *CBL5* gene confers the osmotic or drought stress tolerance in plants [17]. However, the function of the *AtXTH10* gene is currently unknown, so its function was investigated in detail. An overexpressing vector (35S::AtXTH10-eGFP) was constructed and transgenic plants (AtXTH10-OE) were obtained. T-DNA insertion mutant (SALK_1235, named AtXTH10m) of this gene was obtained from the ABRC (Arabidopsis Biological Resource Center, http://abrc.osu.edu). Fluorescence confocal microscopy revealed that most of the AtXTH10 protein was localized in the cell wall (Figure 3A). AtXTH10-OE plants presented a 48.6% increase in root length compared with the wild type (Col), whereas the mutation of this gene led to about a one-third reduction in root length (Figure 3B), suggesting that this gene is involved in root development. Microstructural differences in the root epidermal cells were also observed in the three plants (Col, AtXTH10-OE, and AtXTH10m) by scanning electron microscope. The arrangement of the root epidermal cells in the AtXTH10m mutant was disordered, and the root cells were shorter than those of the wild type (Figure 3C). The arrangement of root epidermal cells in AtXTH10-OE plants was more regular and their root cells were longer than those of wild type plants (Figure 3C). These results indicate that the *AtXTH10* gene affects growth and development of the roots by modifying the cell wall structure.

### 3.4. Prediction of the Root Development Signaling Pathway Involved with the AtXTH10 Gene

**Figure 4:**
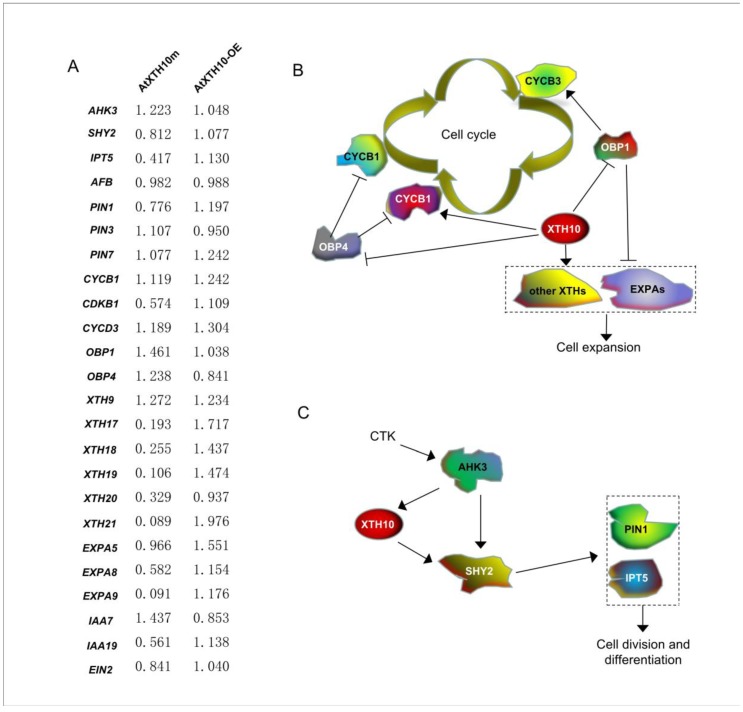
Relative expression levels of the known key genes of root development signal pathway in AtXTH10m and AtXTH10-OE compared with wild type Arabidopsis (Col) plants (**A**). The signal pathway of root cell expansion mediated by cell cycle (**B**). The signal pathway of cytokinin-mediated root division and differentiation (**C**). The graph (**A**) is automatically generated by BAR HeatMapper Tool (http://bar.utoronto.ca) and the depth of color represents the difference of expression. Three biological repeats were performed for this RT-qPCR analysis. Their average relative expression data are marked on the graph.

Cell wall remodeling during plant growth is accompanied by a change in cell volume and number. Breakage and regeneration of cell wall xyloglucans play an important role in cell wall remodeling [42]. XTH is an important cell wall modification enzyme that catalyzes the cleavage and regeneration of xyloglucan [19]. Moreover, some key genes that affect root development have been investigated in Arabidopsis (see Methods). To elucidate whether the *AtXTH10* gene is also involved in the root growth and development signaling pathways and consequently associated with key genes expression patterns, RT-qPCR was used to detect expression levels of 24 genes in wild type (Col), mutant (AtXTH10m), and overexpressing (AtXTH10-OE) plants (Figure 4A). The results indicated that the *AtXTH17*, *AtXTH18*, *AtXTH19*, and *AtXTH21* genes showed similar expression patterns in these three plants, which were downregulated in the mutant AtXTH10m plants and upregulated in the AtXTH10-OE plants. These results suggest that AtXTH10 may function upstream of these genes (Figure 4B). Plant expansin proteins are responsible for cell wall loosening during cell growth, fruit softening, and tube elongation [43]. Mutations or overexpression of the *AtXTH10* gene decreased or increased transcriptional levels of the *EXPA5*, *EXPA8*, and *EXPA9* genes, respectively, suggesting that *AtXTH10* may participate in cell wall remodeling upstream of these genes (Figure 4B). Moreover, the cell cycle regulator (CDKB1) had the highest activity during cell division. When the *AtXTH10* gene was mutated, the transcription level of *CDKB1* was reduced, and overexpression of the *AtXTH10* gene increased the transcription level of this gene, indicating that both regulate cell division (Figure 4B). In addition, the *AtXTH10* gene inhibited expression of the OCS element binding factor binding protein 1 and 4 (*OBP1* and *OBP4*) genes during root development, which are involved in the control of cell division and regulation of the cell cycle [44,45].

One study showed that *AHK3*, *SHY2*, and *IPT5* are involved in the cytokinin (CTK) signaling pathway during root cell division and differentiation [46]. CTK regulates its receptor gene *AHK3*, and then *AHK3* regulates its downstream *SHY2* gene. Similarly, *SHY2* also regulates expression of the *IPT5* gene [46]. This study reported that the expression level of the *AHK3* gene increases in *AtXTH10* mutant plants, while the expression levels of *SHY2* and *IPT5* decreased. The *SHY2* and *IPT5* genes were upregulated in AtXTH10-OE plants, while the expression level of *AHK3* was similar to that of the wild type. This result suggests that *AHK3* may regulate the *AtXTH10* gene, thereby affecting expression of the *SHY2* and *IPT5* genes. That is, the *AtXTH10* gene was located downstream of the *AHK3* and upstream of the *SHY2* and *IPT5* in this signaling pathway (Figure 4C). Auxin regulates growth and development in plants. Polar transport and distribution of auxin transport carrier genes play important roles in the development of root apices [47]. Next, the expression patterns of three auxin transport protein genes (*PIN1*, *PIN3*, and *PIN7*) were examined, and only the *PIN1* gene had a significant expression change in these three plants. This result indicates that *PIN1* may be regulated by the *AtXTH10* gene and plays a major role in the development of roots (Figure 4C). In this study, the expression pattern of the *indole-3-acetic acid inducible 19* (*IAA19*) gene was also investigated and its expression profiles were similar to those of *XTH* and *EXPA,* as described above. However, the expression profiles of the *IAA7* gene contrasted to those of *IAA19* in the wild type, mutant, and overexpressing plants. Perhaps, *AtXTH10* plays a negative role regulating function of the *IAA7* gene. All of these results suggest that IRSs could play crucial roles in growth and developmental processes in plants.

## 4. Conclusions

In this study, 1343 IRS sequences were identified in the Arabidopsis genome, and more than 30% of the structures were located in the promoter regions. A deletion analysis of the IRSs was performed in detail on the CR6 and CR12 promoters. The results indicated that the CR6 and CR12 IRSs play positive and negative roles in promoter activity, respectively, suggesting that they may contribute to regulatory function. Next, the function of the *AtXTH10* gene containing the CR6 promoter was also investigated in detail. Transgenic and mutant plants analysis showed that *AtXTH10* affected root growth and development by regulating cell wall structure. Moreover, some key genes involved in root development were also verified to be affected by the *AtXTH10* gene. This finding suggests that plant IRSs play crucial roles in regulating gene expression that consequently affect plant growth and developmental processes.

## Supplementary Materials

The following are available online at https://www.mdpi.com/2223-7747/8/5/130/s1. Table S1: All identified IRSs and their locations in Arabidopsis genome. Table S2: Primers used for construction of expression vectors and transgenic plant analysis. Table S3: Primers used for RT-qPCR in this study. Figure S1: Location of T-DNA insertion in the mutant (SALK_1235).
