# Supplementary figures and images for: Interspaced Repeat Sequences Confer the Regulatory Functions of AtXTH10, Important for Root Growth in Arabidopsis
Source: Plants (Basel). 2019 May 16;8(5):130. doi: 10.3390/plants8050130 (PMC6572656; doi:10.3390/plants8050130)

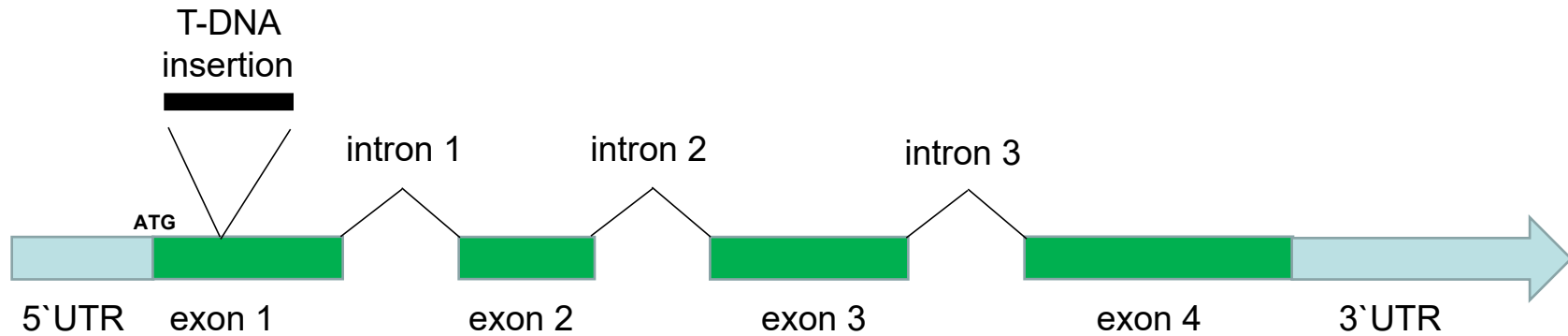

Supplement: Supplementary file 1 [file plants-08-00130-s001.zip › plants-488267-supplementary-revised 2/Figure S1.pdf]
