# Supplementary material for: Interspaced Repeat Sequences Confer the Regulatory Functions of AtXTH10, Important for Root Growth in Arabidopsis
Source: Plants (Basel). 2019 May 16;8(5):130. doi: 10.3390/plants8050130 (PMC6572656; doi:10.3390/plants8050130)
Supplement: Supplementary file 1 [file plants-08-00130-s001.zip › plants-488267-supplementary-revised 2/Table S2.doc]

**Table S2.** Primers used for construction of expression vectors and transgenic plant analysis.

| **Primer names** | **Primer sequences (5`--3`)** | **Ta(℃)** | **Size (bp)** | **Uses** |
| --- | --- | --- | --- | --- |
| CR6-1 | AAGCTTTTCTTTGGATTGAATAAATCCCCA | 56 | CR6-1/CR6-2: 712 | Clone and deletion of CR6 promoter |
| CR6-2 | GGATCCTTAAGATGTTTGAGGTTGAGTACGAT |  |  |  |
| CR6-4 | GGATCCATGAAAAGTGTTTAA | 52 | CR6-1/CR6-4: 639 |  |
| CR6-6 | GGATCCATATATAACGATCATTGA | 53 | CR6-1/CR6-6: 597 |  |
| CR6-8 | GGATCCTGTGTTTAAAGTAAAT | 52 | CR6-1/CR6-8: 556 |  |
| CR12-1 | AAGCTTGAAGAAGAAGTTTAAATTTGCT | 56 | CR12-1/CR12-2: 647 | Clone and deletion of CR12 promoter |
| CR12-2 | GGATCCTTTACTATATCAATATATATTTCA |  |  |  |
| CR12-a | AAGCTTAAATACTTGAATTGT | 52 | CR12-a/CR12-2: 594 |  |
| CR12-b | AAGCTTAATACTTTGTTGT | 51 | CR12-b/CR12-2: 541 |  |
| CR12-c | AAGCTTGCAAATACTATTGT | 52 | CR12-c/CR12-2: 486 |  |
| CR12-3 | AAGCTTCTCATTGGACAGCCCA | 52 | CR12-2/CR12-3: 450 |  |
| AtXTH10-F | TCTAGAATGACTCTAATAAATCG | 51 | AtXTH10-F/AtXTH10-R: 897 | Clone of AtXTH10 gene |
| AtXTH10-R | CCCGGGATATTTAGGAAGAGAAC |  |  |  |
| 35S-1 | GGAAGGTGGCTCCTACAAAT | 52 | 35S-1/AtXTH10-R: 1155 | Identification of transgenic plants |
