# Supplementary material for: Interspaced Repeat Sequences Confer the Regulatory Functions of AtXTH10, Important for Root Growth in Arabidopsis
Source: Plants (Basel). 2019 May 16;8(5):130. doi: 10.3390/plants8050130 (PMC6572656; doi:10.3390/plants8050130)
Supplement: Supplementary file 1 [file plants-08-00130-s001.zip › plants-488267-supplementary-revised 2/Table S3.doc]

**Table S3.** Primers used for RT-qPCR in this study.

| **Gene** | **Accession ID** | **Forward primer** | **Reverse primer** | **Ta (℃)** | **E%** | **R^2^** | **Size (bp)** |
| --- | --- | --- | --- | --- | --- | --- | --- |
| *AHK3* | NM_102494 | TTGTTGCACATATAATCCACGC | GTGGCAAGGAACTGTGAC | 60 | 93.9 | 0.999 | 128 |
| *SHY2* | NM_001331470 | ATGGTGATTGGATGCTCATT | AGCCTAAACCTTTGGCTTC | 60 | 89.4 | 0.996 | 103 |
| *IPT5* | NM_001343584 | CGTCCACGACACTTACGAA | TTGGAACCACCGGCTATG | 60 | 86.5 | 0.996 | 115 |
| *AFB* | NM_124377 | TTTGATTGAGTCTGAGGTCACG | AATTGATAGGAGCTTCCACAC | 60 | 97.7 | 0.999 | 114 |
| *PIN1* | NM_106017 | CTCCGAGACCTTCCAACTAC | CACCACTTCCTCCAGATTGATA | 60 | 89.1 | 0.996 | 109 |
| *PIN3* | NM_105762 | GGCTCCGAATCCAGAGTT | TTGGAAGAGTCGTCTGCT | 60 | 92.6 | 0.998 | 105 |
| *PIN7* | NM_001084115 | AAATCGGTGACGATGGTAAG | TTAGACGGACGAGGAGTCATA | 60 | 90.2 | 0.998 | 107 |
| *CYCB1* | NM_119913 | ATGTTGAGAATGACCTCGC | GAGATGCCATGTAATCTCGTG | 60 | 94.1 | 0.999 | 103 |
| *CDKB1* | NM_115278 | AGGACCTAATCCTAAGCCTC | GGTGAAGCACACCATGACTA | 60 | 93.5 | 0.996 | 102 |
| *CYCD3* | NM_119579 | CATCAGTAGTTGCCGCAG | CTTGGTTAAGTTAAGGACACCG | 60 | 97.2 | 0.999 | 108 |
| *OBP1* | NM_114901 | CTTCCATGTCCTCGTTGTG | GTCCAGTAACGACGACAAG | 60 | 96.4 | 0.999 | 108 |
| *OBP4* | NM_125478 | GGATCGAGTGGTCGACGTTA | CCCGAATCCAAACGGTGTA | 60 | 95.8 | 0.999 | 114 |
| *XTH9* | NM_116559 | GACCAATATCTACGTGAACGG | ACTCCAGAGGATTGAGTAAGT | 60 | 93.4 | 0.996 | 101 |
| *XTH17* | NM_001334201 | ACAATGTCGATGGATGTGTATG | CATGTATTTACTTTGCACACCT | 60 | 80.4 | 0.991 | 126 |
| *XTH18* | NM_119174 | CCAGGAGTTTCTCTATGGCAA | CAATCTCATCCCACGTAGT | 60 | 83.6 | 0.994 | 117 |
| *XTH19* | NM_119175 | TATCAGCGTCTATGCAGGC | CAGAGGATTTGTCAAGCGAG | 60 | 86.2 | 0.992 | 120 |
| *XTH20* | NM_124181 | CTTTGCAGCACAATATGAGC | CGAGAGAGAAAGAAGATTTCCG | 60 | 89.5 | 0.994 | 119 |
| *XTH21* | NM_127436 | ACTCAAGGCAAGGGTGAT | AGAACACTATGTGACTCGGA | 60 | 80.1 | 0.991 | 107 |
| *EXPA5* | NM_113824 | CGTACACTCGGTCTCAATGAA | TTGTGGTAACAACAAACGACAG | 60 | 90.5 | 0.996 | 126 |
| *EXPA8* | NM_129623 | GTACACGCCGTCTCAATCA | GGCTTTGGTCGTTCATGTAT | 60 | 89.8 | 0.992 | 107 |
| *EXPA9* | NM_120304 | CCCATCAATTCTTATCACCGC | CGAGATCAAAGTGCTCACGA | 60 | 78.6 | 0.994 | 105 |
| *IAA7* | NM_113205 | CAGTTGAGAGTCCTGCCA | AACGGAGCCTTCTTTGTTAG | 60 | 94.3 | 0.999 | 102 |
| *IAA19* | NM_112422 | CCTTGAAAGATGGTGACAACT | AACCTCTTGCATGACTCTAGAA | 60 | 90.8 | 0.998 | 116 |
| *EIN2* | NM_112777 | TGGACGAATCTAGAGTCGTG | CCGACATCAAAGCTACGG | 60 | 93.8 | 0.999 | 101 |
| *ACTIN2* | NM_112764 | GTATGTCGCCATCCAAGCTGTT | TCAGTAAGGTCACGTCCAGCAA | 60 | 96.2 | 0.999 | 244 |

Ta: annealing temperature; E%: primer efficiency; R^2^: co-efficient of determination.
